# Preservice mathematics teachers’ perceptions of mathematical problem solving and its teaching: A case from China

**DOI:** 10.3389/fpsyg.2022.998586

**Published:** 2022-11-03

**Authors:** Peijie Jiang, Yong Zhang, Yanyun Jiang, Bin Xiong

**Affiliations:** ^1^School of Mathematics and Statistics, Hunan Normal University, Changsha, China; ^2^School of Mathematics, Yunnan Normal University, Kunming, China; ^3^The High School Attached to Hunan Normal University, Changsha, China; ^4^School of Mathematical Sciences, East China Normal University, Shanghai, China; ^5^Shanghai Key Laboratory of Pure Mathematics and Mathematical Practice, Shanghai, China

**Keywords:** pre-service teacher, perceptions, problem solving, mathematics teaching, in-service teacher

## Abstract

Preservice mathematics teachers’ accurate understanding of mathematical problem solving and its teaching is key to the performance of their professional quality. This study aims to investigate preservice mathematics teachers’ understanding of problem solving and its teaching and compares it with the understanding of in-service mathematics teachers. After surveying 326 in-service mathematics teachers, this study constructs a reliable and valid tool for the cognition of mathematical problem solving and its teaching and conducts a questionnaire survey on 26 preservice mathematics teachers. Survey results reveal that preservice mathematics teachers have a good understanding of mathematical problem solving and its teaching and are more confident in the transfer value of problem solving ability. By contrast, in-service teachers are more optimistic that problem solving requires exploration, continuous thinking, and the participation of metacognition. This article concludes that preservice mathematics teachers should focus more on the initiative and creativity of students and put students at the center of education. In addition, teacher educators should provide more teaching practice opportunities for preservice teachers. The findings also show that in-service teachers’ understanding of problem solving and its teaching is inferior to that of preservice teachers on some indicators, implying the importance of post-service training for in-service teachers.

## Introduction

Mathematics learning is essential in cultivating the philosophical thinking and rational spirit of students (Jiang and Xiong, 2021a). Many countries, including China, attach great importance to mathematics education (Jiang and Xiong, 2021b). Problems are the heart of mathematics. Mathematics is developed and perfected by constantly solving various problems, and the mathematician’s main reason for existence is to solve problems (Mason, 2016). Therefore, mathematics consists of problems and solutions. Learning how to solve mathematical problems is at the heart of mathematics education (Polya, 2002a). Solving mathematical problems helps students better understand mathematics (Munzar et al., 2021). To a large extent, mathematics education cultivates the problem solving abilities of students (Polya, 1990). Learning to solve problems and think mathematically requires continuous reflection on the nature of this activity (Arcavi et al., 1998). Therefore, the problem solving teaching ability of mathematics teachers is vital. Nowadays, the relevance of problem solving in teaching and learning mathematics has become commonplace (Libedinsky and Soto-Andrade, 2016).

When teachers invite students to solve a problem, they do not know as much about how to solve the problem as many people think (Andrews and Xenofontos, 2015). Improving problem solving requires focusing on some recommendations, mainly for teachers and their education (Zimmermann, 2016). Teachers’ knowledge of teaching content affects their classroom practice, which involves student learning and achievement (Peterson et al., 1989). Problem solving is getting from where you are to where you want to be by continuously reformulating the problem until it becomes something you can manage (Kilpatrick, 2016). The cognition of mathematical problem solving affects mathematical problem solving and teaching behavior. Teachers’ beliefs about mathematics impact their teaching, and teachers with different views about mathematics teach differently (Philipp, 2007; Lester and Cai, 2016). Teachers are central to advancing the affective atmosphere and social interaction of the class (Pehkonen et al., 2016), and their beliefs have a considerable impact on the nature of classroom practice (Kayan Fadlelmula and Cakiroglu, 2008). Therefore, teachers need to have a good understanding of mathematical problem solving and its teaching.

Preservice mathematics teachers are prospective teachers who will teach mathematics after graduation (Jiang and Jiang, 2022). Many preservice teachers complete advanced mathematics courses with a limited interpretation of critical terms, incorrect beliefs about the nature of mathematics, and a failure to recognize that mathematics stimulates analytical thinking and creativity (Paolucci, 2015). They have difficulty raising and solving problems (Işık and Kar, 2012; Mallart et al., 2018). Teacher education can alleviate negative attitudes or beliefs about mathematics and teaching mathematics to college students preparing to become teachers (Looney et al., 2017). Preservice mathematics teachers with proper training will have better problem solving and problem solving teaching performance (Crespo and Sinclair, 2008; Karp, 2010). They need a teacher preparation program that focuses their attention on the learning of the students they are teaching (Kilpatrick, 2016). They must understand mathematics, teaching, and pedagogy (Register et al., 2022). Proper understanding of mathematical problem solving and its teaching is an essential part of the professional quality of preservice mathematics teachers and can effectively guide their future teaching of mathematical problem solving.

Significant advances have been made in understanding the affective, cognitive, and metacognitive aspects of problem solving in mathematics and other disciplines (Lester and Cai, 2016). Research on the correlation between the use of various problem solving strategies and problem solving success has been plentiful over the last century (Schoenfeld, 2007), and there have been many suggestions for teaching problem solving effectively (Mason, 2016). However, empirical studies of preservice teachers’ understanding of mathematical problem solving and its teaching are still rare. The big question facing current mathematical problem solving research and teaching practice is this: How do we make meaningful problem solving a regular feature of mathematics classrooms (Leong et al., 2016)? It is necessary to train many outstanding mathematics teachers, and a feasible method to achieve this is to pay attention to the education of preservice teachers. We should understand the mathematical problem solving and teaching knowledge of preservice teachers. On the basis of this knowledge, we can develop educational strategies to improve their problem solving teaching skills.

Improving the mathematical problem solving teaching ability of teachers requires understanding the mathematical problem solving and teaching perception of in-service and preservice teachers. For high-quality mathematics (problem solving) teaching, preservice teachers think that “developing students’ thinking ability” and “mathematical communication ability” is more critical. By contrast, in-service teachers think “learning arrangement” and “building connections” are more important (Clooney and Cunningham, 2017). Age and work experience may shape beliefs related to mathematical problem solving (Metallidou, 2009). It is helpful for the training of preservice teachers to understand how in-service teachers view mathematical problem solving and its teaching. Therefore, comparing the cognition of preservice and in-service teachers toward mathematical problem solving and its teaching is necessary. The cognition of preservice teachers can be better understood by placing them in the background of in-service teachers’ perceptions. Issues such as whether their perceptions have something in common, what the differences are, how to narrow the gap, how to further optimize their perceptions, which perceptions can be optimized before they are employed, and which can only be optimized afterward are not only significant for the training of preservice teachers but also help the continuing training of in-service ones.

Mathematical problem solving teaching in China is relatively successful, and Chinese students perform very well in international mathematics competitions, such as the International Mathematical Olympiad (Xiong and Jiang, 2021). A study of 495 Chinese preservice mathematics teachers showed that their beliefs about mathematics teaching are most correlated with their inquiry-based teaching practices (Yang et al., 2020). However, as Cai and Nie (2007) pointed out, mathematical problem solving research in China has been much more content- and experience-based than cognitive- and empirical-based. Empirical studies of problem solving related to preservice mathematics are scarce. Therefore, China’s experience is worth examining. The present study aims to answer the following questions:

1. What do preservice mathematics teachers know about mathematical problem solving and its teaching?
2. What is the difference between preservice and in-service mathematics teachers’ cognition of mathematical problem solving and its teaching?

Establishing a model and related tools to study preservice teachers’ understanding of mathematical problem solving has important implications for future work in mathematics education and teacher education.

## Literature review

Mathematical problem solving was once a hot research issue in mathematics education (Lester, 1994; Schoenfeld, 2007). The content covered in the literature review below includes mathematical problem solving, preservice mathematics teachers, and problem solving for preservice mathematics teachers. This section expounds on the background and starting point of this study from these three aspects.

### Mathematical problem solving

Problems generally refer to stimulating situations that cannot be responded to with ready-made responses and require that specific barriers be overcome between the given information and the goal. Mathematical problems can generally be divided into problems for construction and problems to prove (Polya, 1945). Schoenfeld (1985) pointed out that mathematical problems are usually divided into two categories: the common practice problem and the problem that students must experience before solving.

George Polya, the founder of the mathematical problem solving theory, described problem solving as follows (Polya, 1962, p. v): “Solving a problem means finding a way out of a difficulty, a way around an obstacle, attaining an aim which was not immediately attainable.” In general, solving problems that require effort and exploration improves one’s thinking skills. Polya’s problem solving table provides a perspective of the problem solving process from four stages: *Understanding the Problem, Devising a Plan, Carrying out the Plan*, and *Looking Back* (Rosiyanti et al., 2021). What Polya proposed was a heuristic approach, and his theories of mathematical problem solving were far-reaching.

Lester (1994) systematically reviewed the research on mathematical problem solving in terms of research content and methods from 1970 to 1984. He summarized the current state of problem solving research and recommended future studies. Schoenfeld (2013) proposed a decision theory based on his four-factor framework for mathematical problem solving. Research on mathematical problem solving is vibrant. The connotation and requirements of mathematical problem solving ability are also constantly changing. There have been many research results on the technology of mathematical problem solving, creativity in mathematical problem solving, and emotion and aesthetics in mathematical problem solving (Amado et al., 2018). In terms of research methods, the research methods of mathematical problem solving include differential analysis, thinking aloud, correlation analysis, and teaching experiments (Bao and Zhou, 2009).

Problem solving is a significant learning activity, but the quality of problem solving teaching needs to be improved. As Lester and Charles (1992) once pointed out, research on mathematical problem solving provides little specific information on problem solving learning. The role of the teacher in teaching is neglected, and little attention is paid to what happens in real classrooms. The research focuses on the individual, on the theory but not on the class, which is the shortcoming of current problem solving teaching research. Lester (2013) reflected on the study of mathematical problem solving teaching and made four assertions: (1) We need to rethink what mathematical problems and problem solving are. (2) We know very little about how to improve students’ metacognition through problem solving. (3) Mathematics teachers need not be problem solving experts but must be serious problem solving students. (4) Mathematical problem solving is not always a high-level cognitive activity.

It is worth mentioning that problem solving is a core activity in Chinese mathematics teaching and learning. Mathematical problem solving activities in China have the following characteristics: (1) high level of reasoning, often involving multi-step and complex formal mathematical reasoning; (2) high comprehensive knowledge, with general mathematical problems involving multiple knowledge points; (3) high operation requirements, including high symbolic calculus ability; (4) simple background, in which more emphasis is placed on the connections within mathematics; and (5) presence of various solutions and high problem solving skills. The problem solving characteristics mentioned above are affected by examinations and courses on the one hand and classroom teachings on the other, such as emphasizing “Bianshi” teaching, question-type training, and reduction methods (Bao, 2017).

In conclusion, despite the many research results, we still know very little about how to develop the metacognitive abilities of students through the teaching of mathematical problem solving. The role of teachers in problem solving learning has been neglected. To improve the problem solving ability of students, we must pay attention to the part of teachers. The problem solving teaching ability of mathematics teachers is also crucial. In addition, some empirical research supported by exact facts and data is necessary to study mathematical problem solving teaching.

### Preservice mathematics teachers

Increasing attention is being paid to the training of preservice mathematics teachers. Preservice teachers refer to those who want to become teachers. In this study, preservice mathematics teachers are “quasi” mathematics teachers, including undergraduate students in Mathematics education and master’s students in curriculum and teaching theory or mathematics teaching (Tong and Yang, 2018; Jiang and Jiang, 2022). The research on preservice mathematics teachers consists of studies on the current situation, curriculum and instruction, skill training, and teaching knowledge research. Many researchers have studied the mathematical knowledge and related beliefs of preservice mathematics teachers as well as their curriculum and teaching knowledge (Pamuk and Peker, 2009; Prescott et al., 2013; Dede and Karakus, 2014; Paolucci, 2015; Lutovac, 2019). Some researchers agree that preservice mathematics teachers must be better prepared for future mathematics teaching (Land et al., 2015; Lau, 2021).

In many countries, preservice mathematics teachers are taught mathematics and mathematics pedagogy in the mathematics department and education department of their universities. The training of preservice mathematics teachers requires interdisciplinary cooperation (Beers and Davidson, 2009; Hodge, 2011; Goos and Bennison, 2018). Some researchers pointed out that the teaching skills of preservice mathematics teachers need to be strengthened (Özgen and Alkan, 2014; Land et al., 2015; Gokalp, 2016). Many researchers (Llinares and Valls, 2009; Özmantar et al., 2010; Baki et al., 2011; Hechter et al., 2012; Caniglia and Meadows, 2018; Saralar et al., 2018) are also concerned about the ability of preservice mathematics teachers to use Information and Communication Technology (ICT).

Compared to their Western counterparts, Chinese preservice mathematics teachers are more familiar with traditional mathematics thinking and teaching and are not competent in TPACK (Xiang and Ning, 2014). After more than 100 years of development, China’s formal preservice teacher education has formed some unique models and characteristics (Tong and Yang, 2018). The training program for preservice mathematics teachers in China has two distinct features (Li et al., 2008). The first is that it lays a solid mathematical foundation for normal students to have a higher mathematical literacy. The second is that it pays attention to the review and research of elementary mathematics because everyone believes that a deep understanding of elementary mathematics and solid problem solving ability are fundamental to becoming qualified middle school mathematics teachers. Under the test-oriented education tradition, qualified teachers must have high problem solving skills.

However, many questions about preservice mathematics teachers have not been effectively addressed. The study of how to train preservice mathematics teachers efficiently remains an essential topic in education worldwide.

### Problem solving of preservice mathematics teacher

Although some studies do reveal the characteristics of preservice mathematics teachers in problem solving, such studies are not rich enough. Preservice mathematics teachers have many deficiencies in problem solving. In terms of problem solving skills, most preservice mathematics teachers have low problem solving skills (Özgen and Alkan, 2012). For instance, regarding questioning skills, preservice mathematics teachers commit seven types of errors in asking questions about fractional splitting (Işık and Kar, 2012). Some preservice mathematics teachers have difficulty asking questions about everyday life, fitting into the school curriculum at a given educational level, and posing questions that students can self-correct (Mallart et al., 2018). Preservice mathematics teachers can employ problem solving strategies and problem solving, but their use of different techniques is limited (Avcu and Avcu, 2010). Likewise, they have difficulty expressing operations in the mathematical language (Özdemir and Çelik, 2021). Thus, they need a better understanding of problem solving and its teaching through teaching practice.

Preservice mathematics teachers have difficulty choosing specific mathematical problems, and it is rather difficult for them to find unconventional mathematical problem situations (Temur, 2012). Therefore, many problems in problem solving teaching for preservice mathematics teachers need to be discussed in depth. Zsoldos-Marchis (2015) studied the effectiveness of collaborative problem solving in changing the attitudes of preservice elementary teachers toward mathematics. They found that students who used cooperative learning methods had statistically significant positive changes in their enjoyment of mathematics. The students improved their belief in the usefulness of mathematics, preferring to solve unconventional problems. Other researchers explored the learning process of preservice mathematics teachers taking part in a middle school mathematics methods curriculum, noting the need for further research on such programs (Gómez, 2009). Some researchers have tried to paint a picture of problem solving in Chinese mathematics education, revealing the knowledge of Chinese preservice mathematics teachers in problem solving and teaching (Cai and Nie, 2007), but the research is far from extensive. To summarize, research on problem solving and problem solving teaching for preservice mathematics teachers is necessary. Evidence from classrooms, students, and frontline teachers is significant.

## Materials and methods

### Research methods

#### Methods

A study in Turkey used a survey research method to understand how Turkish preservice primary school mathematics teachers perceive problem solving in mathematics education (Kayan Fadlelmula and Cakiroglu, 2008). The current study adopted the questionnaire survey method as it can be used to investigate the knowledge of Chinese mathematics teachers on mathematical problem solving and its teaching. First, the researchers designed a preliminary questionnaire based on the literature and then examined the problem solving and teaching knowledge of in-service mathematics teachers through the online teaching and research community (WeChat and QQ) to develop research tools and establish norms. The online survey instrument is *WENJUANXING* (a widely used platform for publishing online questionnaires in China). The researchers presented links to the questionnaires through WeChat and QQ, and in-service mathematics teachers willing to participate in the survey could click the links to fill out the questionnaires. The teachers were fully informed of the purpose of the study, and filling out the online questionnaire was voluntary. Data generated by in-service teachers served as the norm and could be used to develop and validate the validity of research tools. Structural modeling was used to deal with the data. After the research tools were formed, representative preservice mathematics teachers were selected to conduct a questionnaire survey. According to research ethics requirements, the questionnaire survey was conducted only after the preservice mathematics teachers signed the informed consent.

#### Participants

Chinese mathematics teachers attach great importance to problem solving, must solve many problems, and regard problem solving as one of the most critical teaching tasks (Xiong and Jiang, 2021). This study is part of a more extensive study. In the larger study, the researchers explored how to design curriculum instruction to facilitate the development of problem solving instructional skills among preservice mathematics teachers. Therefore, it is essential to specify the sample for the study. A total of 199 in-service mathematics teachers effectively participated in the online survey, most of whom came from high schools of good quality in Guangxi, China. Therefore, they could serve as representatives of excellent teachers in the province. As they joined the online mathematics teaching and research group independently, these teachers could be considered as having a high interest in mathematics education. They already have some teaching experience and all have been engaged in the teaching of mathematical problem solving. In addition, 127 mathematics competition coaches from all over China also completed the questionnaire online. Chinese mathematics competition coaches have high problem solving skills and problem solving teaching skills.

The participants in this study came from a local key normal university in China. A total of 26 full-time first-year graduate students, 2 males and 24 females, were selected. Two majored in mathematics curriculum and teaching theory and 24 majored in mathematics teaching. They are preservice mathematics teachers and will all be engaged in mathematics teaching after graduation. Most of them have studied mathematics courses such as Mathematical Analysis, Advanced Algebra, Modern Algebra, Functions of Real Variables, Functions of Complex Variables, and Topology at the undergraduate level. They have studied practical courses such as Mathematics Instructional Design and Teaching Skills Training and have experience in micro-teaching and educational practice. However, they are still students and do not have enough teaching experience yet.

#### Procedure

Questionnaire surveys are one of the most popular data-gathering methods in the social sciences. This study demonstrates the development and use of questionnaires according to the purpose of the study. The basic process of this study was as follows: conduct literature review→design research methods→conduct online surveys on in-service mathematics teachers→design survey tools based on online surveys→conduct surveys on preservice mathematics teachers→collect and analyze data→report results. The study first investigated the problem solving and teaching cognition of in-service mathematics teachers. The researchers developed valid questionnaires based on literature and data on in-service teachers. Then they contacted participants to ask if they would like to participate. With the participants’ consent, the researchers committed to protecting their privacy, distributed the electronic questionnaires to them, and technically assisted them in completing the questionnaires. After the participants completed the questionnaires, the researchers retrieved the data. Once the data of preservice mathematics teachers were collected, they were compared with the data of in-service mathematics teachers.

#### Data collection and analysis

As mentioned earlier in section “Methods,” this study collected data through the Internet and surveyed preservice mathematics teachers by using questionnaires. Therefore, the research data can reveal the knowledge of in-service and preservice teachers about mathematical problem solving and its teaching. After the original network data were collected, the data were preprocessed to obtain valid data. The researchers carried out structural equation modeling based on the valid data and then designed a survey tool for preservice mathematics teachers. Descriptive statistics were carried out on the data of both in-service and preservice mathematics teachers, and the similarities and differences between the teachers were compared. The researchers then performed inferential statistics to arrive at more general conclusions. This study used SPSS 22.0 and AMOS 22.0 to process the data. AMOS 22.0 was used to build a structural equation model for understanding mathematical problem solving and its teaching. The maximum likelihood method was used to estimate the model.

### Research tools

Beliefs are part of the affective domain of an individual that influences the learning process (Manderfeld and Siller, 2019). Understanding young students’ emotional factors and beliefs about mathematics is a complex task (Giaconi et al., 2016), as is understanding teachers’ perceptions of mathematical problem solving and its teaching. Many research frameworks have been built on mathematical beliefs (Hannula, 2011, 2012). The teaching beliefs of mathematics teachers refer to their orientations toward teaching mathematics, which involve perspectives regarding instructional activities, the cognitive processes of students, and the purpose of mathematics (Wang et al., 2022). Currently, the transmissive and constructive taxonomy of teaching beliefs is commonly used in existing studies and international assessments, such as TEDS-M (Blömeke and Kaiser, 2014). Unfortunately, the framework for teachers’ perception of problem solving and its teaching is relatively rare.

In this research, understanding mathematical problem solving and its teaching refers to the knowledge and essential viewpoints about mathematical problem solving and its teaching. It is the overall reflection of individual minds on mathematical problem solving and teaching. This study characterizes preservice mathematics teachers’ cognition of mathematical problem solving and its teaching from three aspects: overall impression of mathematical problem solving, specialized knowledge, and teaching perspective. In particular, impression is the perceptual image of problem solving in the individual’s mind, specialized knowledge refers to the individual’s rational understanding of problem solving, and teaching perspective is the attitude and orientation of problem solving teaching. The preparation of measurement items is mainly based on students’ typical mathematical beliefs and Polya’s theories on mathematical problem solving and teaching (Polya, 1945). It is refined and synthesized according to research needs.

Designing a questionnaire means creating valid and reliable questions that address the research objectives, placing them in proper order, and selecting an appropriate administration method. The design of the corresponding measurement items is mainly based on the five-factor cognitive framework of Schoenfeld and is refined and synthesized according to research needs (Schoenfeld, 2016). For example, the five items s8–s12 in the measurement tool correspond to Schoenfeld’s five-factor cognitive framework for mathematical problem solving (the coding and content of the item will be mentioned below). Typical mathematical beliefs held by some of the students mentioned by Schoenfeld above are shown in Table 1, and these beliefs are imperfect.

**TABLE 1:** Typical student beliefs about the nature of mathematics (Schoenfeld, 1992).

Code	Belief
1	Mathematical problems have one and only one correct answer.
2	There is only one correct way to solve any mathematical problem—usually the rule the teacher has most recently demonstrated to the class.
3	Ordinary students cannot expect to understand mathematics because they wish to memorize it and apply what they have learned mechanically but without understanding.
4	Mathematics is a solitary activity done by individuals in isolation.
5	Students who have understood the mathematics they have studied will be able to solve any assigned problem in five minutes or less.
6	The mathematics learned in school has little or nothing to do with the real world.
7	The formal proof is irrelevant to the processes of discovery or invention.

Take the teaching viewpoint of mathematical problem solving as an example. Two items are set up to reflect the orientation of preservice mathematics teachers to the teaching of mathematical problem solving. The two items are “Problem solving requires independent thinking; try not to let students discuss with one another” and “Teaching students to solve problems is to tell students the solution.” Since problem solving requires independent thinking, cooperative learning promotes problem solving (teaching). Teaching students to solve problems introduces students to problem solving and inspires them to think. The formulation of the above two items is not considered the correct understanding.

Each item is set on a five-point Likert scale: strongly disagree, disagree, neutral, agree, strongly agree. There are positive and negative items. The score is based on the correctness of the options. Each option is assigned the order of 1, 2, 3, 4, 5, and each option of the reverse item is given the order of 5, 4, 3, 2, 1. The options of the positive item are assigned as 1, 2, 3, 4, and 5 in turn, and the options of the negative item are designated as 5, 4, 3, 2, and 1. The higher the score, the higher the understanding of mathematical problem solving and teaching. Of course, the so-called correct refers to the point of view with a specific basis, in line with the primary trend.

The entire measurement questionnaire initially included 20 measurement items, and after expert judgment removed 8 duplicate items, the final questionnaire consisted of 12 items. The minimum score of the questionnaire is 12 points and the maximum is 60 points. Considering the characteristics of the five-point-option Likert scale, to better analyze the data, the cognitive level is divided into four grades according to the score, of which 48–60 is graded A, 36–47 is graded B, 24–35 is graded C, and 12–23 is graded D.

According to the score characteristics, the researchers set B and above as the qualification level to present results clearly, with A set as an excellent level. Table 2 shows the number of measurement items and the coding of the three secondary indicators of understanding mathematical problem solving and its teaching.

**TABLE 2:** Problem solving and its teaching cognition measurement framework.

Target	Indicators	Number of items	Code
Cognition	Overall impression	4	s1, s2, s3, s4
	Specialized knowledge	6	s7, s8, s9, s10, s11, s12
	Teaching perspective	2	s5, s6

The items of the entire questionnaire are as follows:

• s1. Problems in problem solving refer to the exercises in textbooks, which generally have conventional and mechanized solutions.
  A. Strongly disagree    B. Disagree    C. Neutral    D. Agree    E. Strongly agree
• s2. Problem solving is calculating the result according to the conventional algorithm.
  A. Strongly disagree    B. Disagree    C. Neutral    D. Agree    E. Strongly agree
• s3. The four problem solving stages are understanding the problem, making a plan, implementing the plan, and reviewing. After getting the correct answer, you don’t have to spend too much time on the retrospective stage.
  A. Strongly disagree    B. Disagree    C. Neutral    D. Agree    E. Strongly agree
• s4. Problem solving is a skill that has little meaning after a student graduates.
  A. Strongly disagree    B. Disagree    C. Neutral    D. Agree    E. Strongly agree
• s5. Problem solving requires independent thinking; try not to let students discuss with one another.
  A. Strongly disagree    B. Disagree    C. Neutral    D. Agree    E. Strongly agree
• s6. Teaching students to solve problems is to teach students ready-made solutions.
  A. Strongly disagree    B. Disagree    C. Neutral    D. Agree    E. Strongly agree
• s7. Problem solving generally requires mobilizing mathematical thinking methods through exploration, association, and reasoning.
  A. Strongly disagree    B. Disagree    C. Neutral    D. Agree    E. Strongly agree
• s8. A good problem solver must have a good knowledge structure.
  A. Strongly disagree    B. Disagree    C. Neutral    D. Agree    E. Strongly agree
• s9. Emotional attitudes have an important impact on problem solving.
  A. Strongly disagree    B. Disagree    C. Neutral    D. Agree    E. Strongly agree
• s10. Excellent problem solvers can constantly adjust their thinking to solve problems.
  A. Strongly disagree    B. Disagree    C. Neutral    D. Agree    E. Strongly agree
• s11. Good problem solvers use heuristics well and experiment with different strategies.
  A. Strongly disagree    B. Disagree    C. Neutral    D. Agree    E. Strongly agree
• s12. Excellent problem solvers have a good sense of experience.
  A. Strongly disagree    B. Disagree    C. Neutral    D. Agree    E. Strongly agree

## Results

### Reliability and validity

The cognitive questionnaire for mathematical problem solving and teaching is based on literature, and its content validity has been discussed above. A total of 203 questionnaires were returned, of which 199 were valid questionnaires. Since the questionnaire has 12 items, the ratio of the number of valid questionnaires returned to the number of items in the questionnaire exceeds 10:1, which can ensure the validity of the model significance test (Jiang et al., 2022).

The Cronbach’s alpha coefficient of the questionnaire is 0.770. The internal consistency is good because the questionnaire has only 12 items. The KMO value of the questionnaire is 0.798, which makes it suitable for factor analysis. From Table 3, the average score for the test teachers (199 mathematics teachers) is 47.73; they are in the B class but very close to the A class. Their intermediate perception of problem solving and teaching is above passable and very close to good. Their minimum score is 33 (C), a failing knowledge level. Their highest score is 60, with a standard deviation of 5.356. The upper quartile is 51, the median is 48, and the lower quartile is 45. The perceptions of problem solving and teaching of half of the teachers reached an excellent level, and the understanding of problem solving and teaching of more than 75% of the teachers was above the qualified group.

**TABLE 3:** Basic statistics for the recognition of in-service teachers.

Statistics	Value
Average	47.73
Median	48.00
Standard deviation	5.356
Minimum	33
Max	60
Upper quartile	51.00
Lower quartile	45.00

**FIGURE 1:**
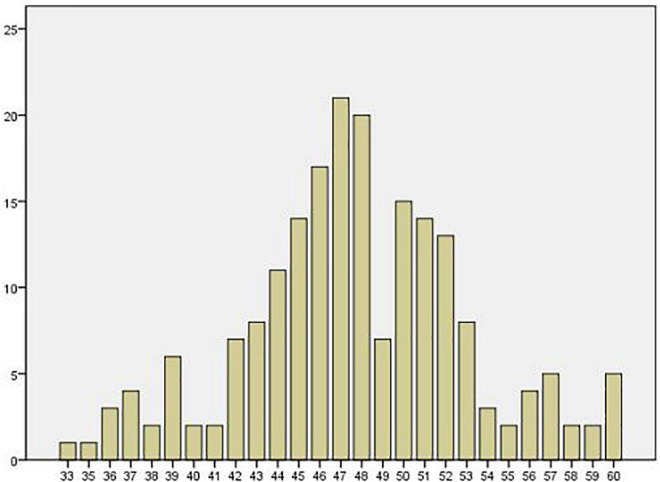
Bar graph of in-service teacher scores.

Figure 1 illustrates the frequency distribution of scores, which is approximately an inverted bell. The distribution of scores reflects the characteristics of a normal distribution, and most scored between 43–53. Although the views of in-service mathematics teachers are not necessarily correct, their knowledge of problem solving and its teaching is considered representative. Their views can represent mathematics teachers’ relatively proper understanding of problem solving and its teaching. The data of the test teachers provide a reference standard for problem solving and their teaching knowledge level, which can be compared with the subsequent analysis of preservice mathematics teachers.

The chi-square value of the original model after modification is 45.185, and the significance probability value is *p* = 0.589 > 0.05. The model is in good agreement with the sample data. Among them, RMR value is 0.027 < 0.050, GFI value is 0.966 > 0.900, AGFI value is 0.944 > 0.900, PGFI value is 0.594 > 0.500, and NFI value, RFI value, IFI value, TLI value and CFI value are all greater than 0.900. Therefore, the model is adapted.

**FIGURE 2:**
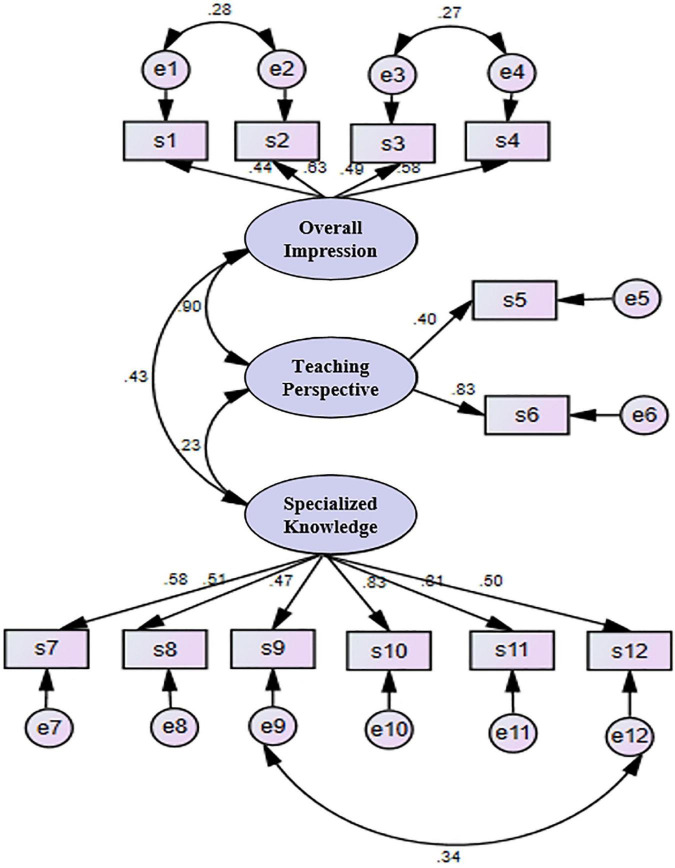
Cognitive model of problem solving and its teaching.

Since factor loadings in the range of 0.30–0.40 are considered to meet the minimum requirements for explanatory structure (Hair et al., 2009), as shown in Figure 2, most factor loadings in the model are higher than 0.50. In addition, only four factor loading values are lower than 0.50 but higher than 0.40. Although factor loading values greater than 0.50 are generally considered to be of practical significance, the factor loading values of this model all reached the acceptable minimum requirements.

In the above model, the correlation between errors has practical significance because there is a correlation between the corresponding items. Routine and mechanics in item s1 have the same meaning as the regular algorithm in item s2, though the former emphasizes what a problem is. By contrast, the latter emphasizes problem solving, but the two are related. Items s3 and s4 are mainly related in that these two viewpoints are refuted by Polya, the founder of mathematical problem solving theory. The two viewpoints often appear simultaneously and are widely known. The correlation between items s9 and s12 is mainly reflected in their emphasis on non-intellectual factors.

In this study, the questionnaire survey data of 127 problem solving experts were added to the original data of 199 in-service primary and secondary school teachers. These data were obtained through questionnaires initiated by the national middle school mathematics competition coaches on WeChat and QQ groups. This study conducted a confirmatory factor analysis on the questionnaire based on these 326 data.

The results showed that the Cronbach’s alpha coefficient of the questionnaire is 0.757, and the reliability of the questionnaire is acceptable. The KMO value of the questionnaire is 0.810, and the questionnaire has good structural validity. The chi-square value of model fit obtained by maximum likelihood estimation is 63.277, and with the significance probability value *p* = 0.069 > 0.05, the model works the sample data. On the indicators of model adaptation, RMR value is 0.027 < 0.050, GFI value is 0.969 > 0.900, AGFI value is 0.949 > 0.900, PGFI value is 0.596 > 0.500, and NFI value (0.947), RFI value (0.928), IFI value (0.987), TLI value (0.982), and CFI value (0.987) are all greater than 0.900. Thus, all values meet the standard of model fitting.

Except for two factor loading values in the model lower than 0.5 (0.42 and 0.45), the other factor loading values are not lower than 0.5, thus meeting the minimum model adaptation requirements. Overall, the questionnaire has acceptable reliability, content validity, and construct validity and can be used to investigate the level of problem solving and teaching knowledge among preservice mathematics teachers.

The above 199 in-service primary and secondary school teachers represent general primary and secondary school mathematics teachers as well. Their data provide a reference standard for mathematical problem solving and teaching cognition for this study.

The single-sample Kolmogorov–Smirnov normality test of the scores of each item and the total score of the 199 middle school teachers in the test are shown in Table 4. The distribution of the scores of each item and the total score of the test teachers does not obey the normal distribution. The subsequent test of the score should use a non-parametric test.

**TABLE 4:** One-sample Kolmogorov–Smirnov normality test.

	s1	s2	s3	s4	s5	s6	s7	s8	S9	s10	s11	s12	Total
p	0.00	0.00	0.00	0.00	0.00	0.00	0.00	0.00	0.00	0.00	0.00	0.00	0.005
Sig.	*	*	*	*	*	*	*	*	*	*	*	*	*

*Significant at the 0.05 level (two-tailed).

Table 5 presents the cognition of the in-service teachers. The following “more than half” (coded as D) means the proportion is in the interval [50%, 60%), “majority” (coded as C) indicates the ratio is in the interval [60%, 75%), “most” (coded as B) means the proportion is in the range [75%, 90%), and “almost all” (coded as A) represents the proportion is in the interval [90%, 100%).

**TABLE 5:** Cognition of in-service teachers.

	s1	s2	s3	s4	s5	s6	s7	s8	s9	s10	s11	s12
Cognition (%)	64.3	60.8	70.2	83.4	60.8	60.8	94.5	93.0	82.4	97.5	98.0	84.0
Code	C	C	C	B	C	C	A	A	B	A	A	B

For items s1, s2, s3, s5, and s6, the majority of the test teachers reached the correct understanding level; for items s4, s9, and s12, most of the test teachers reached the right understanding level; and for items s7, s8, s10, and s11, almost all teachers tested reached the level of proper cognition.

### Descriptive analysis

Twenty-six questionnaires were sent out in this study and 26 were returned. Therefore, all 26 were valid questionnaires. Recovered data were coded and analyzed using SPSS22. The Cronbach’s alpha coefficient of the questionnaire is 0.741, and considering that the questionnaire only had 12 items, the reliability of the questionnaire is acceptable. The average score of the participants is 48.08 points. The intermediate of the participants’ cognition of mathematical problem solving and teaching reached a reasonable level. The lowest score is 32, highest is 54, standard deviation is 4.30, upper quartile is 51, median is 48, and lower quartile is 47. Half of the participants understood mathematical problem solving and teaching well. More than 75% of the participants have an understanding above the qualified level and close to the excellent level. One participant has a cognition score of 32, which is not in the qualifying group.

It can be seen from Table 6 that most preservice mathematics teachers have a correct understanding of items s1, s2, s3, s5, s6, s8, and s12. Almost all preservice mathematics teachers have reached the right understanding level for s4, s7, s9, s10, and s11.

**TABLE 6:** Cognition of preservice mathematics teachers.

	s1	s2	s3	s4	s5	s6	s7	s8	s9	s10	s11	s12
Percentage	80.7	88.5	84.6	92.3	65.3	69.2	92.3	88.4	98.3	96.2	100	88.4
Code	B	B	B	A	C	C	A	B	A	A	A	B

The specific options for preservice mathematics teachers are shown in Table 7, which presents their views on each item. Most participants favor collaborative problem solving and believe that teaching students to solve problems is not just about giving them solutions. Most participants felt that common practice problems in textbooks have a different meaning than mathematical problems and that problem solving is not the process of practicing regular algorithms. They attach great importance to the role of problem solving retrospectives and believe that retrospectives are a crucial stage of problem solving. They think that a good knowledge structure is an essential foundation for problem solving and that proper problem solving exercises are necessary. Almost all participants believe that the problem solving process requires mathematical thinking and logical methods and that strategies are crucial in the mathematical problem solving process. They pay attention to emotion and attitude in problem solving, affirm the critical role of metacognitive monitoring and regulation in problem solving, and believe that problem solving skills still have value after graduation.

**TABLE 7:** Percentage of specific options for preservice mathematics teachers (%).

	s1	s2	s3	s4	s5	s6	s7	s8	s9	s10	s11	s12
Strongly Agree	3.8	0.0	0.0	0.0	3.8	7.7	11.5	34.6	19.2	34.6	26.9	11.5
Agree	3.8	3.8	3.8	3.8	7.7	11.5	80.8	53.8	73.1	61.5	73.1	76.9
Neutral	11.5	7.7	11.5	3.8	23.1	11.5	3.8	7.7	7.7	3.8	0.0	1.5
Disagree	69.2	80.8	65.4	42.3	61.5	57.7	3.8	3.8	0.0	0.0	0.0	0.0
Strongly Disagree	11.5	7.7	19.2	50.0	3.8	11.5	0.0	0.0	0.0	0.0	0.0	0.0

### Difference analysis

The skewness of preservice mathematics teachers (−2.19) was higher than that of in-service mathematics teachers (−0.23), and their scores were skewed to the left. Their average score (48.08) was slightly higher than that of in-service mathematics teachers (47.73). Their overall awareness of mathematical problem solving and teaching was higher than that of in-service mathematics teachers. The maximum score of the preservice mathematics teachers (54) was lower than that of the in-service mathematics teachers (60), and their minimum score (32) was lower than that of the test teachers (33). Still, their scores were less volatile (standard deviation: 4.30 < 5.36). Preservice mathematics teachers (51.00, 48.00) were the same as in-service mathematics teachers (51.00, 48.00) in the upper quartile and median scores. However, in the lower quartile, their scores (47.00) were slightly higher than those of in-service mathematics teachers (45.00). Preservice mathematics teachers had a slightly better distribution of scores.

In the scores and total scores of s1, s2, s3, s4, and s9, preservice mathematics teachers were higher than in-service mathematics teachers. They scored lower than in-service mathematics teachers on other items. The scores of each item of preservice and in-service mathematics teachers were concentrated around 4 points. The scores of each item of in-service mathematics teachers were concentrated explicitly in 2, 3, 4, and 5. By contrast, the scores of each item of preservice mathematics teachers were concentrated explicitly in 3, 4, and 5 points. It can also be seen that the distribution of preservice mathematics teachers’ scores was good.

The distribution of preservice mathematics teachers’ scores did not meet the normality requirement (Kolmogorov–Smirnov normality test, *p* = 0.00 < 0.05). To describe the differences in cognitive level between the subjects and the test teachers in more detail, a non-parametric independent sample test (Mann–Whitney U rank-sum test) was used to analyze the issues. The score distribution of the teacher was tested, and the results are shown in Table 8. Asymptotic significance (two-tailed) is shown in the table at a significance level of 0.05.

**TABLE 8:** Non-parametric independent samples tests (Mann–Whitney *U* test).

	s1	s2	s3	s4	s5	s6	s7	s8	s9	s10	s11	s12	Total
P	0.484	0.066	0.101	0.030	0.790	0.857	0.019	0.414	0.412	0.040	0.008	0.211	0.316
Sig.				*			*			*	*		

*Significant at the 0.05 level (two-tailed).

There was no statistically significant difference in total scores between preservice and in-service mathematics teachers (*p* = 0.316 > 0.05). Their scores for s1, s2, s3, s5, s6, s8, s9, and s12 were not significantly different (*p* = 0.484, 0.066, 0.101, 0.790, 0.857, 0.414, 0.412, 0.211). There were significant differences in their scores for s4, s7, s10, and s11 (*p* = 0.030, 0.019, 0.040, 0.008).

On item s4, “Problem solving is a skill of little significance after students graduate,” preservice teachers scored significantly higher than did in-service teachers. They were more convinced that problem solving skills still work after graduation.

On item s7, “Problem solving generally requires mobilizing mathematical thinking methods to go through the process of exploration, association, and reasoning,” the scores of preservice teachers were significantly lower than those of in-service teachers, and preservice teachers failed to fully realize that mathematical problem solving is multi-dimensional mathematical thinking that requires the participation in exploration and inquiry. On item s10, “Excellent problem solvers can constantly adjust their thinking to solve problems,” preservice teachers scored significantly lower than did in-service teachers. They did not fully recognize the metacognitive monitoring and regulating effect of mathematical problem solving. On item s11, “Excellent problem solvers use heuristics well and experiment with different strategies,” preservice teachers scored significantly lower than did in-service teachers on their awareness of the use of heuristics and strategies in problem solving.

There was no significant difference between preservice and in-service teachers in their knowledge of mathematical problem solving and its teaching. Still, preservice teachers scored slightly higher on average. Preservice teachers are more aware that problem solving skills are still valid after graduation. At the same time, they know that they need to participate in mathematical thinking, actively explore, mobilize corresponding strategies, and apply metacognitive monitoring and adjustment in mathematical problem solving and its teaching.

## Discussion

First, this study builds a research framework, develops research tools based on existing literature, and tests the reliability and validity of the research framework and research tools. The researchers then use the developed research tools to investigate the mathematical problem solving and teaching perceptions of preservice mathematics teachers and compare the survey results with those of in-service teachers. Finally, the researchers discuss the data results.

Preservice mathematics teachers recognize that problem solving skills are transferable and remain helpful after they graduate. They believe that solving problems requires a process of exploration and effort and fully affirm the importance of emotional attitude in problem solving. They think good problem solvers can constantly adjust their thinking and direction to solve problems as well as use heuristics and other strategies. Problem solving training for preservice mathematics teachers is the core content of mathematics teacher education (dos Santos Morais, 2020). This idea may benefit the good mathematical problem solving and teaching knowledge of preservice teachers. Preservice teachers have positive beliefs about solving math problems, and their views are consistent with the current movement for reform in mathematics education (Kayan Fadlelmula and Cakiroglu, 2008). At the same time, the lack of awareness displayed by in-service teachers relative to preservice mathematics teachers means they need on-the-job training to avoid getting lost in their teaching practice. Some research results point out that some in-service teachers have an insufficient understanding of mathematics teaching, which negatively impact their mathematics teaching (Setoromo and Hadebe-Ndlovu, 2020). The behaviors and attitudes of teachers toward teaching and learning and their knowledge base are the results of the influence of on-the-job training (Ramatlapana, 2009). The participation of in-service teachers in training can facilitate their communication with their peers, help them obtain new information, and update their understanding (Izci and Göktas, 2017). Teacher educators can improve the effectiveness of in-service teacher training by teaching content knowledge orientation and stimulating collaboration among teachers (Selter et al., 2015).

Preservice mathematics teachers also understand the meaning of problems and problem solving, the importance of problem solving review, knowledge structure, and practical problem experience. They recognize the importance of independent thinking for problem solving but ignore the value of collaborative problem solving. Compared with in-service teachers, some preservice teachers think that teaching students to solve problems equates to telling students ready-made solutions. However, as Polya advises mathematics teachers, the best way for students to learn mathematics is to discover it independently (Polya, 1945). Preservice teachers must focus more on the initiative and creativity of students and put them at the center (Polya, 2002b). Many teachers are usually in the early stages of their careers, teaching in ways that are not consistent with their beliefs about teaching (Beswick, 2012). If they want to connect belief to practice, then they need to think about practice (Schoenfeld, 2003). They can change their beliefs about mathematics and its teaching by focusing on how students learn and think about mathematics (Hough et al., 2006). Teachers obtain knowledge by reflecting on the goal accomplishment, learning process, and thinking approach of the students; the efficiency of media; and the recommendation of experts (Sudejamnong et al., 2014). Hence, preservice teachers need to acquire further knowledge in teaching practice. It is not enough that they know what to do but do not know why. Preservice mathematics teachers need to understand the nature of educational philosophy.

Research indicates that many preservice teachers show anxiety about teaching mathematics (Steele et al., 2013) and that teacher training programs have little effect on their beliefs (Dede and Karakus, 2014). However, the results of the current study show no significant difference between preservice and in-service mathematics teachers in their mathematical problem solving and teaching knowledge. Preservice teachers are more confident of the transfer value of problem solving ability, while in-service teachers are more confident that problem solving requires exploration and continuous thinking. According to Polya (2002), the founder of the problem solving theory, teaching students to solve problems introduces them to thinking. The problem solving process is a constant thinking process. Preservice teachers may need to experience more problem solving practices and problem solving teaching practices to appreciate this concept more deeply. They should have the opportunity in their studies to solve (and pose) appropriate problems of similar—sometimes also more demanding—types they use as a teacher later on at school (Zimmermann, 2016). The education and professional development of preservice mathematics teachers span diverse backgrounds, each of which legitimizes different perspectives on mathematics teaching (Ramdhany et al., 2018). Learning by experience is of essential importance in everyday life and the academic field (Fritzlar, 2016). Preservice mathematics teachers need to learn in practice and summarize their experiences independently.

The cognitive framework for mathematical problem solving and its teaching proposed in this paper is valid. Its reliability and validity have been tested and can be used for research. The findings provide a framework for studying preservice mathematics teachers’ understanding of problem solving and its teaching. The research results confirm that preservice mathematics teachers understand mathematical problem solving and teaching well. Furthermore, knowing the similarities and differences in understanding between preservice and post-service teachers will help train preservice mathematics teachers better. The findings of this study confirm that the education of preservice mathematics teachers in China, especially at the postgraduate level, is effective in the perception of problem solving teaching (Li et al., 2008). At the same time, the education of preservice teachers should strengthen teaching practices to increase their experience in dealing with practical teaching problems. Every study has its limitations, and this research is no exception. Specifically, the research framework is too simple, the items do not involve an understanding of ICT, the number of items in the questionnaire is too small, and the sample is from only one provincial key normal university. Therefore, the conclusions of this study are not generally applicable. Scientific and careful sampling can minimize the bias caused by selection (Walters, 2021). In addition, teachers with different levels of professional knowledge have different pedagogical focuses on the functions and beliefs of mathematics and demonstrate different teaching methods (Zhang, 2022). Drawing implications for educational practice from the comparison of preservice and in-service mathematics teacher in this study is not straightforward. Future studies should design a richer and more in-depth research framework, design survey tools with higher reliability and validity, and adopt more scientific sampling methods.

## Conclusion

Compared with in-service mathematics teachers, preservice mathematics teachers pursuing postgraduate studies have a good understanding of mathematical problem solving and its teaching. Preservice and in-service teachers share much of the same cognition toward mathematical problem solving and its teaching. From the situation of 26 preservice mathematics teachers, it is believed that the training of China’s postgraduate-level preservice mathematics teachers has been successful. The difference is that preservice teachers acquire this knowledge through school learning while in-service teachers form more understanding through teaching practice. Moreover, the post-employment knowledge of in-service teachers may cover some of the correct knowledge when they were preservice teachers, which means that on-the-job training throughout their entire career is essential. Preservice mathematics teachers develop well in the cognition that can be established through theoretical study but are insufficient in the knowledge that can only be found through practice. Therefore, more practical opportunities must be created for them. These opportunities to practice include educational traineeships and internships but should not be limited to this, extending toward, for example, consideration of the learning opportunities ICT provides.

## Data availability statement

The original contributions presented in this study are included in the article/supplementary material, further inquiries can be directed to the corresponding authors.

## Ethics statement

The studies involving human participants were reviewed and approved by Human Subject Protection Committee of East China Normal University. The patients/participants provided their written informed consent to participate in this study.

## Author contributions

PJ was the primary author of this research. YZ reviewed the manuscript and made suggestions for revision. YJ analyzed and processed the data for this research. BX gave the overall guidance for this research. All authors contributed to the article and approved the submitted version.
